# Sacubitril-Valsartan in Patients Requiring Hemodialysis

**DOI:** 10.1001/jamanetworkopen.2024.29237

**Published:** 2024-08-20

**Authors:** Dustin Le, Morgan E. Grams, Josef Coresh, Jung-Im Shin

**Affiliations:** 1 Division of Nephrology, Department of Medicine, Johns Hopkins School of Medicine, Baltimore, Maryland; 2 Division of Precision Medicine, Department of Medicine, New York University, New York, New York; 3 Optimal Aging Institute, Department of Medicine, New York University, New York, New York; 4 Department of Epidemiology, Johns Hopkins Bloomberg School of Public Health, Baltimore, Maryland

## Abstract

**Importance:**

Randomized clinical trials have shown that sacubitril-valsartan reduces the risks of mortality and hospitalization in patients with heart failure with reduced ejection fraction (HFrEF), but patients with kidney failure requiring dialysis were excluded.

**Objective:**

To investigate the comparative effectiveness of sacubitril-valsartan vs angiotensin-converting enzyme inhibitors or angiotensin receptor blockers (ACEIs or ARBs) in patients with HFrEF requiring hemodialysis.

**Design, Setting, and Participants:**

This retrospective, 1:1 propensity score–matched comparative effectiveness study included patients who were 18 years or older with HFrEF, enrolled in Medicare Parts A, B, and D, and survived at least 90 days receiving in-center hemodialysis from July 8, 2015, to December 31, 2020. Patients were excluded for less than 180 days of continuous Medicare Parts A, B, and D primary payer coverage or prior dispensing of sacubitril-valsartan. Data analysis was conducted from September 23, 2023, to June 25, 2024.

**Exposures:**

New use of sacubitril-valsartan vs new or continued use of ACEIs or ARBs.

**Main Outcomes and Measures:**

The associations between initiation of sacubitril-valsartan therapy and all-cause mortality, cardiovascular mortality, all-cause hospitalization, and HF hospitalization were assessed using Cox proportional hazards regression models in a propensity score–matched sample.

**Results:**

Participants included 1:1 matched pairs of 1434 sacubitril-valsartan users and 1434 ACEI or ARB users (mean [SD] age, 64 [13] years). Of the 2868 matched participants, 996 (65%) were male; 987 (34%) were Black or African American and 1677 (58%) were White; and median dialysis vintage was 3.8 (IQR, 1.8-6.3) years. The median follow-up was 0.9 (IQR, 0.4-1.7) years. Sacubitril-valsartan (vs ACEI or ARB) therapy was associated with a reduction in all-cause mortality (hazard ratio [HR], 0.82 [95% CI, 0.73-0.92]) and all-cause hospitalization (HR, 0.86 [95% CI, 0.79-0.93]) but not cardiovascular mortality (HR, 1.01 [95% CI, 0.86-1.19]) or HF hospitalization (HR, 0.91 [95% CI, 0.82-1.02]). There was a decrease in hyperkalemia (HR, 0.71 [95% CI, 0.62-0.81]) and no difference in hypotension (HR, 0.99 [95% CI, 0.83-1.19]). Only 195 participants (14%) ever received the maximum combination dose of sacubitril (97 mg twice daily) and valsartan (103 mg twice daily).

**Conclusions and Relevance:**

In this comparative effectiveness study of patients with HFrEF requiring hemodialysis, sacubitril-valsartan therapy was associated with beneficial effects in all-cause mortality and all-cause hospitalization.

## Key Points

**Question:**

Is the use of sacubitril-valsartan associated with reduced risk of all-cause mortality and hospitalization among individuals requiring hemodialysis for heart failure with reduced ejection fraction (HFrEF)?

**Findings:**

In this comparative effectiveness research study of 1434 patient pairs, initiation of sacubitril-valsartan therapy was associated with a statistically significant 18% reduction in all-cause mortality and 14% reduction in all-cause hospitalization.

**Meaning:**

These findings suggest sacubitril-valsartan therapy may benefit individuals with HFrEF requiring hemodialysis.

## Teaser

This comparative effectiveness study assesses the risk of all-cause mortality, cardiovascular mortality, all-cause hospitalization, and heart failure hospitalization among patients with heart failure with reduced ejection fraction requiring dialysis who receive combined sacubitril-valsartan vs angiotensin-converting enzyme inhibitors or angiotensin receptor blockers.

## Introduction

Cardiovascular (CV) disease causes 50% of deaths in individuals in the US who require kidney replacement therapy, and heart failure (HF) is the most common CV diagnosis, with a prevalence of 40% in this population.^1^ Multiple medical therapies in randomized clinical trials have shown to be effective in reducing CV mortality in HF (such as angiotensin receptor–neprilysin inhibitors [ARNIs], sodium-glucose cotransporter-2 inhibitors, and spironolactone), but patients receiving dialysis were excluded from these trials.^2,3,4^ Thus, studies using observational data in this population are helpful to quantify the effect of medication interventions.

Sacubitril-valsartan, a first-in-class ARNI, was shown to be superior to angiotensin-converting enzyme inhibitors (ACEIs) in the Prospective Comparison of ARNI With ACEI to Determine Impact on Global Mortality and Morbidity in Heart Failure (PARADIGM-HF) trial,^2^ and for those with HF with reduced ejection fraction (HFrEF), initiation of sacubitril-valsartan therapy is a class I recommendation by the American Heart Association, American College of Cardiology, and Heart Failure Society of America guidelines.^5^ In addition to improvements in CV mortality, sacubitril-valsartan has been associated with reductions in all-cause mortality, HF hospitalization, and all-cause hospitalization.^2,6,7^ Despite the high annual price of $5576 for individuals with Medicare Part D,^8^ sacubitril-valsartan has been shown to be associated with overall decreases in health care expenditure in a population not receiving dialysis.^9^ Thus, sacubitril-valsartan may have therapeutic potential with significant clinical implications for patients with HFrEF requiring dialysis. Data regarding clinical benefit, however, are limited, and randomized clinical trials in Asia are ongoing.^10,11^

Two observational studies^12,13^ have examined clinical outcomes with sacubitril-valsartan in patients requiring dialysis, and no mortality benefit was seen. Furthermore, outcomes regarding HF hospitalization were mixed (one showing benefit; the other showing harm). Given limited sample sizes of published data from only Asian populations (range, 7-110 patients with total numbers <400),^12,13,14,15,16,17,18,19^ we sought to examine the effectiveness of sacubitril-valsartan using the US Renal Data System (USRDS). We assessed the risk of all-cause mortality, CV mortality, all-cause hospitalization, and HF hospitalization.

## Methods

### Data Source

The USRDS database^1^ is a national surveillance system that captures most US patients requiring dialysis and kidney transplant. The USRDS includes Medicare Part A, B, and D claims (consisting of hospitalizations, outpatient care, billing diagnoses, and medications dispensed) and Medicare enrollment history, which can be combined with USRDS data. The Johns Hopkins School of Public Health Institutional Review Board approved the study and waived the need for informed consent owing to the use of deidentified registry data. We followed the International Society for Pharmacoeconomics and Outcomes Research (IPSOR) reporting guidelines for comparative effectiveness research (eAppendix in Supplement 1).

### Target Trial Emulation

#### Eligibility Criteria and Study Population

We used the target trial emulation framework comparing new users of sacubitril-valsartan vs new or continued users of ACEIs or angiotensin receptor blockers (ARBs) among patients with HFrEF receiving hemodialysis (eTable 1 in Supplement 1). Enrollment started July 8, 2015 (after the US Food and Drug Administration approval date for sacubitril-valsartan), and participants were eligible at treatment strategy initiation (ie, baseline) if they had a history of HFrEF, were 18 years or older, and survived at least 90 days receiving in-center hemodialysis (per USRDS recommendations).^1^ Heart failure with reduced ejection fraction was ascertained from Medicare records by the presence of at least 1 inpatient or 2 outpatient codes (428.2X from the *International Classification of Diseases, Ninth Revision,* or I50.2X from the *International Statistical Classification of Diseases, Tenth Revision*) any time prior to baseline, which have 97.7% specificity for individuals with HFrEF (<45% in patients not receiving dialysis).^6,20,21^

Exclusion criteria at enrollment consisted of not receiving in-center hemodialysis (eg, kidney recovery without dialysis, kidney transplant, transition to other dialysis modality, or unconfirmed dialysis modality), less than 180 days of continuous Medicare Parts A, B, and D primary payer coverage, prior dispensing of sacubitril-valsartan, and missing baseline information on the Centers for Medicare & Medicaid Services (CMS) Form 2728 (eFigure 1 in Supplement 1).

#### Treatment Strategies

We compared new users of sacubitril-valsartan with new or continued users of ACEIs or ARBs between July 8, 2015, and December 31, 2020. New users of sacubitril-valsartan had 180 days prior to enrollment without a previous dispense for sacubitril-valsartan. Similarly, new users of ACEIs or ARBs had 180 days prior to enrollment without a previous dispense of ACEIs or ARBs, and for continued users of ACEIs or ARBs (dispensed in the previous 180 days), we randomly selected an enrollment date from eligible dispenses.^22^

We included individuals with a history of ACEI or ARB use, as sacubitril-valsartan is a first-in-class medication, and landmark trials of sacubitril-valsartan (PARADIGM-HF and Comparison of Sacubitril-Valsartan vs Enalapril on Effect on NT-proBNP [N-terminal pro–brain-type natriuretic peptide] in Patients Stabilized from an Acute Heart Failure Episode [PIONEER-HF]) enrolled patients with^2^ and without^23^ recent ACEI or ARB use. Prescription of ACEIs or ARBs for US patients receiving hemodialysis is also common^1^ and higher among those with HF.

### Covariate Ascertainment

Patient demographic characteristics (including race), initial dialysis access, comorbidities at dialysis initiation (ie, atherosclerotic heart disease, cancer, chronic pulmonary disease, cerebrovascular disease, diabetes, dysrhythmia, hypertension, myocardial infarction, other cardiac disease, and peripheral vascular disease), smoking status, ESRD (end-stage renal disease or kidney failure) network region (ie, geographic region), and etiology of kidney failure (diabetes or not) were abstracted from CMS Medical Evidence Form 2728. Race was clinician reported from preselected categories that included Black or African American and White. We collapsed American Indian or Alaska Native, Asian, Hawaiian or Other Pacific Islander, Middle Eastern or Arabian, Other, and unknown to a single category of Other due to limited sample size. Certain comorbidities (ie, atherosclerotic heart disease, cerebrovascular disease, peripheral vascular disease, other cardiac disease, respiratory disease, liver disease, dysrhythmia, cancer, and diabetes)^24^ were defined by the presence of 1 inpatient or 2 outpatient *ICD-9* or *ICD-10* claims in the 180 days prior to baseline (eTable 3 in Supplement 1). Number of hospitalizations and primary care visits (defined as office visits to Internal Medicine, Family Practice, or General Medicine) in the previous 180 days were captured in Medicare Part A and B, respectively. We used the most updated body mass index, KT/V (dialyzer clearance of urea × dialysis time divided by volume of distribution of urea [a measure of dialysis adequacy]), and erythropoietin stimulating agent use from dialysis records. Prior medications (<90 days prior to baseline) (eTable 2 in Supplement 1), Medicare Part D subsidy status, dialysis vintage (baseline minus dialysis start date), baseline calendar year, strength of historic ACEI or ARB dose (none, low, medium, or high) (eTable 4 in Supplement 1),^6^ and number of ACEI or ARB dispenses in the past 180 days were also noted. Dose of ACEIs or ARBs was determined by the National Drug Code of medication dispensed.

### Treatment Assignment: Randomization Emulation by 1:1 Propensity Score Match

We used the MatchIt package^25^ to perform nearest neighbor propensity score matching without replacement to identify the 1:1 matched pairs of sacubitril-valsartan and ACEI or ARB users. The propensity score model included all baseline covariates,^26^ and we used exact matching on history of ACEI or ARB use, meaning patients who received ACEIs or ARBs in the 180 days prior to sacubitril-valsartan therapy initiation were matched against ACEI or ARB users who also had ACEI or ARB use in the 180 days prior (ie, continued users of ACEIs or ARB). Similarly, sacubitril-valsartan users without recent ACEI or ARB use were matched against new users of ACEI or ARB therapy.^6^

### Follow-Up, Outcomes, and Causal Contrasts

Individuals were followed up until study outcome, kidney transplant, renal recovery without dialysis, loss of Medicare Primary Payer status, death, or December 31, 2020, whichever came first. The primary study outcome was all-cause mortality, and secondary outcomes included CV mortality, index hospitalization, and index HF hospitalization.

Date and cause of death was determined by the USRDS.^1^ We categorized CV mortality as causes of death that the USRDS determined as arrhythmia, atherosclerosis, cardiac arrest, cardiomyopathy, congestive HF, myocardial, pericardial, or valvular cause of death (CMS Form 2746). During follow-up, index hospitalization was ascertained by the first inpatient billing code date. Index HF hospitalization was abstracted from Medicare Part A in any billing position (eTable 3 in Supplement 1).

For safety outcomes, we examined hyperkalemia and hypotension using either 1 hospitalization claim or 2 outpatient claims (eTable 3 in Supplement 1). For outpatient claims, we used the earlier date. We categorized adverse events as *any* for both outpatient and inpatient claims and *hospitalization* for inpatient claims.

We estimated both intention-to-treat (ITT) and as-treated (AT) effects. In the AT analysis, individuals were additionally censored at end of medication dispense, defined as no refill within 30 days after end date of last dispense. Sacubitril-valsartan users were additionally censored for switching to an ACEI or ARB.

### Statistical Analysis

#### Main Analysis

Data were analyzed from September 23, 2023, to June 25, 2024. Baseline characteristics between sacubitril-valsartan and ACEI or ARB users were compared before and after matching with absolute standardized mean differences (SMDs) less than 0.10 considered good balance.^27^

We used both Cox proportional hazards regression and Poisson regression on the 1:1 matched cohort to assess sacubitril-valsartan therapy initiation and risk of all-cause mortality, CV mortality, all-cause hospitalization, and HF hospitalization. For each outcome, we estimated the hazard ratio (HR), incidence rate ratio, and incident rate difference. We repeated this analysis for the safety outcomes of hyperkalemia and hypotension. Kaplan-Meier plots are shown for all-cause mortality, CV mortality, all-cause hospitalization, and HF hospitalization.

We looked at subgroups by age (≥65 years), sex, race, smoking status, diabetic kidney disease, atherosclerotic heart disease, dysrhythmia, history of diabetes, diuretic use, and dialysis vintage (by quartile) with all-cause mortality and all-cause hospitalization using the 1:1 matched cohort. In each subgroup, we included an interaction term between the treatment variable and subgroup indicator variable in the regression model. We examined the SMDs within subgroups and included variables with SMDs of at least 0.10 as covariates.^28^ Given that we hypothesized there would be a significant interaction with dialysis vintage (with lower vintage associated with more benefit), we report baseline demographics by dialysis vintage.

We also examined sacubitril-valsartan titration patterns after the first dose. Dispensing patterns were defined using the starting, maximum, and final doses. To evaluate for a dose effect, we used multivariable Cox proportional hazards regression (adjusted for all covariates) to examine all-cause mortality and all-cause hospitalization among sacubitril-valsartan users using an ITT approach across starting dose (with 24 and 26 mg, respectively, as reference). Statistical significance was defined by a linear trend across doses. Analyses were performed using the survival package in R, version 4.2.2 (R Program for Statistical Computing)^31^ and Stata, version 17 (StataCorp LLC).^32^ Significant results were determined by a 2-tailed *P* < .05.

#### Sensitivity Analyses

We repeated our primary and secondary outcome analysis using 1:4 matching, and we repeated the AT analysis using a 60-day (instead of 30-day) refill window to define medication discontinuation. To address residual and/or unmeasured confounding, we examined fracture hospitalization as a negative control outcome, as risk of fracture is not expected to differ by sacubitril-valsartan vs ACEI or ARB use, and we estimated the E-value,^29^ which is the minimum relative risk that an unmeasured confounder would need to have with both the exposure and the outcome to explain the observed association between sacubitril-valsartan use and all-cause mortality. To estimate the relative risk of study covariates and all-cause mortality, we used multivariate logistic regression adjusted for all covariates in Table 1. Finally, we repeated the analysis of secondary outcomes using competing risk models to take into account competing risks (non-CV death for CV mortality; all-cause mortality for both hospitalization and safety outcomes [hypotension or hyperkalemia].^30^

**Table 1.** Baseline Characteristics of Sacubitril-Valsartan Users vs ACEI or ARB Users Before and After 1:1 Match^a^

Characteristic	Entire cohort			1:1 Matched cohort		
	Sacubitril-valsartan (n = 1434)	ACEI or ARB (n = 29 654)	SMD	Sacubitril-valsartan (n = 1434)	ACEI or ARB (n = 1434)	SMD
Age, mean (SD), y	64 (13)	65 (13)	0.05	64 (13)	64 (14)	0.05
Sex						
Female	490 (34)	12 667 (43)	0.18	490 (34)	506 (35)	0.02
Male	944 (66)	16 987 (57)		944 (66)	928 (65)	
Race^b^						
Black or African American	500 (35)	11 097 (37)	0.05	500 (35)	487 (34)	0.04
White	840 (59)	16 622 (56)		840 (59)	837 (58)	
Other^c^	94 (7)	1935 (7)		94 (7)	110 (8)	
Year of medication therapy initiation						
2015	<10^d^	6407 (22)	1.08	<10^d^	<10^d^	0.05
2016	80s^d^	6214 (21)		80s^d^	70s^d^	
2017	179 (12)	4801 (16)		179 (12)	188 (13)	
2018	270 (19)	4251 (14)		270 (19)	276 (19)	
2019	464 (32)	4432 (15)		464 (32)	463 (32)	
2020	433 (30)	3549 (12)		433 (30)	433 (30)	
Dialysis vintage, median (IQR), y	3.9 (1.9-6.3)	3.7 (1.8-6.0)	0.05	3.9 (1.9-6.3)	3.5 (1.8-6.3)	0.03
KT/V	1.6 (1.4-1.7)	1.6 (1.4-1.8)	0.06	1.6 (1.4-1.7)	1.6 (1.4-1.7)	0.01
Current smoker	110 (8)	2511 (8)	0.03	110 (8)	108 (8)	0.01
Part D subsidy	978 (68)	21 418 (72)	0.08	978 (68)	1015 (71)	0.05
History of ESA use	1201 (84)	25 288 (85)	0.04	1201 (84)	1198 (84)	0.01
Kidney failure not from diabetes	751 (52)	13 744 (46)	0.12	751 (52)	731 (51)	0.03
US geographic region						
Midwest	302 (21)	7071 (24)	0.09	302 (21)	298 (21)	0.02
Northeast	364 (25)	7194 (24)		364 (25)	356 (25)	
South	522 (36)	9852 (33)		522 (36)	525 (37)	
West	246 (17)	5537 (19)		246 (17)	255 (18)	
Started dialysis via catheter	1109 (77)	22 824 (77)	0.01	1109 (77)	1134 (79)	0.04
BMI, median (IQR)	26 (23-30)	26 (22-31)	0.01	26 (23-30)	27 (23-31)	0.05
Atherosclerotic heart disease	825 (58)	17 708 (60)	0.04	825 (58)	802 (56)	0.03
Cancer	157 (11)	2970 (10)	0.03	157 (11)	172 (12)	0.03
Dysrhythmia	700 (49)	13 535 (46)	0.06	700 (49)	685 (48)	0.02
Cerebrovascular disease	308 (21)	7978 (27)	0.13	308 (21)	303 (21)	0.01
Other cardiac disease	786 (55)	14 216 (48)	0.14	786 (55)	770 (54)	0.02
Diabetes	1023 (71)	22 475 (76)	0.10	1023 (71)	1021 (71)	0.003
History of gastrointestinal tract bleed	171 (12)	3874 (13)	0.03	171 (12)	147 (10)	0.05
Hypertension	1263 (88)	26 527 (89)	0.04	1263 (88)	1273 (89)	0.02
Inability to ambulate	40 (3)	1301 (4)	0.09	40 (3)	50 (3)	0.04
Liver disease	161 (11)	5424 (18)	0.20	161 (11)	161 (11)	<0.001
Peripheral vascular disease	717 (50)	17 315 (58)	0.17	717 (50)	718 (50)	0.001
Respiratory disease	370 (26)	8904 (30)	0.09	370 (26)	376 (26)	0.01
No. of hospitalizations within 6 mo, mean (SD)	1.79 (1.95)	1.93 (2.07)	0.07	1.79 (1.95)	1.78 (1.83)	0.01
No. of primary care visits within 6 mo, mean (SD)	2.25 (3.12)	1.73 (2.53)	0.18	2.25 (3.12)	2.35 (3.60)	0.03
No. of medications <90 d, mean (SD)	10.76 (5.02)	10.91 (4.95)	0.03	10.76 (5.02)	10.72 (5.07)	0.01
Medications <90 d						
Aldosterone antagonist	82 (6)	734 (2)	0.16	82 (6)	82 (6)	<0.001
Antiplatelets	430 (30)	8207 (28)	0.05	430 (30)	417 (29)	0.02
β-blockers	1124 (78)	22 377 (75)	0.07	1124 (78)	1104 (77)	0.03
CCBs	401 (28)	12 829 (43)	0.32	401 (28)	422 (29)	0.03
Diuretics	396 (28)	6879 (23)	0.10	396 (28)	402 (28)	0.01
DOAC	184 (13)	1809 (6)	0.23	184 (13)	198 (14)	0.03
GLP-1RA	14 (1)	113 (8)	0.07	14 (1)	12 (1)	0.01
Insulin	351 (24)	8235 (28)	0.08	351 (24)	352 (25)	0.002
Phosphate binder	796 (56)	17 236 (58)	0.05	796 (56)	846 (59)	0.07
SGLT2	<10^d^	<10^d^	0.03	<10^d^	<10^d^	<0.001
Warfarin	132 (9)	3390 (11)	0.07	132 (9)	137 (10)	0.01
Prior ACEI or ARB use						
Low dose	235 (16)	4470 (15)	0.24	235 (16)	232 (16)	0.01
Moderate dose	271 (19)	7039 (24)		271 (19)	270 (19)	
High dose	190 (13)	5736 (19)		190 (13)	194 (14)	
No use	738 (51)	12 409 (42)		738 (51)	738 (51)	0.01
No. of ACEI or ARB refills within 6 mo, mean (SD)	1.28 (1.84)	1.80 (2.30)	0.18	1.28 (1.84)	1.28 (1.88)	0.004

Abbreviations: ACEI, angiotensin-converting enzyme inhibitor; ARB, angiotensin receptor blocker; BMI, body mass index (calculated as the weight in kilograms divided by the height in meters squared); CCB, calcium channel blocker; DOAC, direct oral anticoagulant; ESA, erythropoietin-stimulating agent; GLP-1RA, glucagonlike peptide–1 receptor agonist; KT/V, dialyzer clearance of urea × dialysis time divided by volume of distribution of urea (a quantitative measurement of dialysis adequacy); SGLT2, sodium-glucose cotransporter-2 inhibitor; SMD, absolute standardized mean difference.

^a^ Unless otherwise indicated, data are expressed as No. (%) of participants. Percentages have been rounded and may not total 100.

^b^ Race was ascertained via Centers for Medicaid & Medicare Services Medical Evidence Form 2728 (completed at dialysis initiation).

^c^ Other race was defined as American Indian or Alaska Native, Asian, Hawaiian or Other Pacific Islander, Middle Eastern or Arabian, Other, and unknown.

^d^ Cells with fewer than 10 participants were censored per US Renal Data System reporting policy or rounded down (if multiple categories).

## Results

### Patient Characteristics

**Figure 1.**
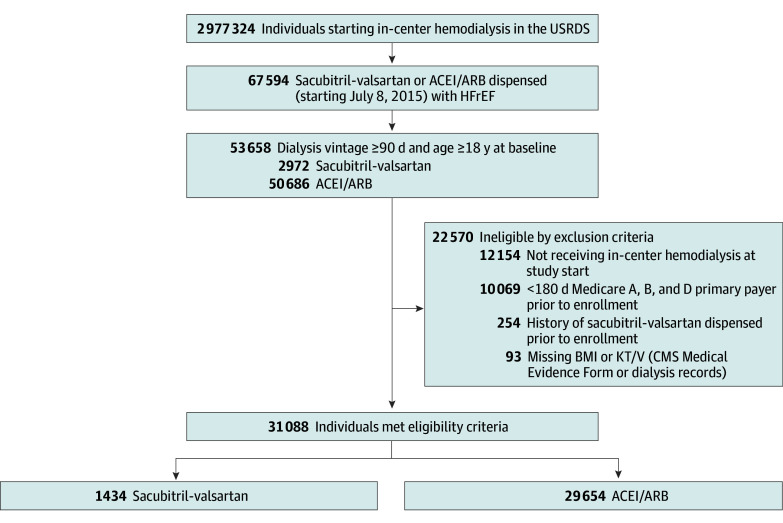
Study Flow Diagram Medication dispensed history and heart failure with reduced ejection fraction (HFrEF) were ascertained using Medicare records. Dialysis vintage was calculated at the time of medication therapy initiation. ACEI indicates angiotensin-converting enzyme inhibitor; ARB, angiotensin receptor blocker; BMI, body mass index; CMS, Centers for Medicare & Medicaid Services; KT/V, dialyzer clearance of urea × dialysis time divided by volume of distribution of urea (a quantitative measurement of dialysis adequacy); and USRDS, US Renal Data System.

We identified 1434 sacubitril-valsartan and 29 654 ACEI or ARB users who met study criteria for a total cohort of 31 088 participants (Figure 1), of whom 17 931 (58%) were male, 13 157 (42%) were female, and mean (SD) age was 65 (13) years. In terms of race, 11 597 participants (37%) were Black or African American, 17 462 (56%) White, and 2029 (7%) Other race. Prior to matching, sacubitril-valsartan users (compared with ACEI or ARB users) were more likely to be male, have nondiabetic kidney disease, be seen by their primary care physician, use aldosterone antagonists, and use direct oral anticoagulants. They were less likely to have atherosclerotic heart disease, history of transient ischemic attack or stroke, diabetes, recent hospitalization, peripheral vascular disease, liver disease, respiratory disease, recent calcium channel blocker dispensed, and recent ACEI or ARB dispensed (696 [48%] vs 17 245 [58%]). After 1:1 matching, all variables were balanced with SMDs of less than 0.10 (Table 1 and eFigure 2 in Supplement 1).

In the matched cohort of 2868 participants, mean (SD) age of the study population was 64 (13) years; 996 (35%) were female and 1872 (65%) were male; 987 (34%) were Black or African American, 1677 (58%) were White, and 204 (7%) were Other race. Median dialysis vintage was 3.8 (IQR, 1.8-6.3) years and 1386 (48%) had kidney failure due to diabetes (Table 1).

### Follow-Up and Outcomes in the Matched Cohort

**Figure 2.**
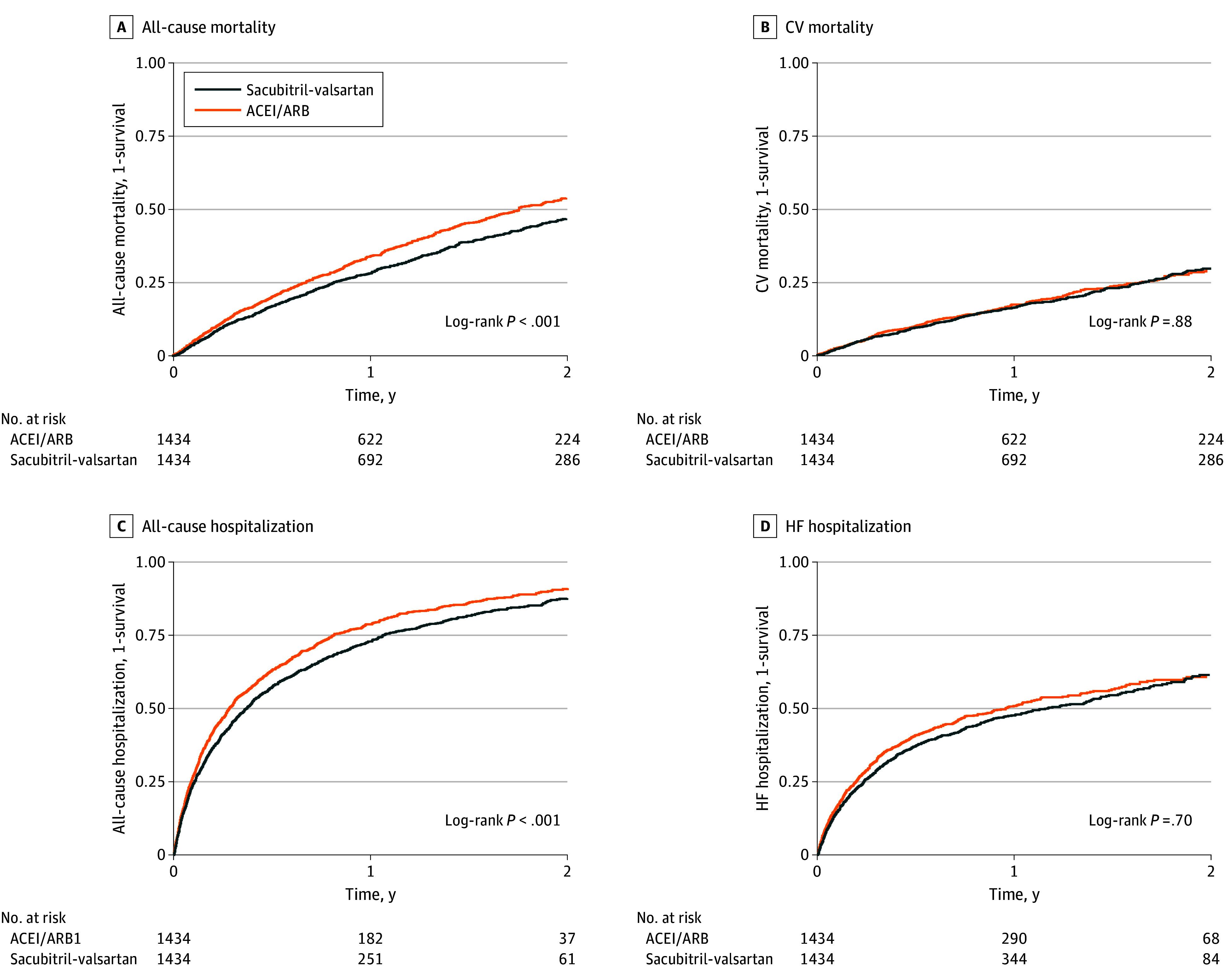
Kaplan-Meier Curves for Primary and Secondary Outcomes Participants were eligible for enrollment from 2015 to 2020, and analysis used an intention-to-treat approach. ACEI indicates angiotensin converting enzyme inhibitor; ARB, angiotensin receptor blocker; CV, cardiovascular; and HF, heart failure.

The median follow-up was 0.9 (IQR, 0.4-1.7) years. The primary outcome of death occurred in 554 individuals (39%) receiving sacubitril-valsartan and in 618 (43%) receiving ACEIs or ARBs. The cumulative incidence of mortality was 28% at 1 year and 46% at 2 years for the sacubitril-valsartan group vs 34% and 53%, respectively for the ACEI or ARB group (Figure 2). Compared with ACEIs or ARBs, sacubitril-valsartan use was associated with a lower risk of all-cause mortality (HR, 0.82 [95% CI, 0.73-0.92]) and all-cause hospitalization (HR, 0.86 [95% CI, 0.79-0.93]). There was no association with CV mortality (HR, 1.01 [95% CI, 0.86-1.19]) or HF hospitalization (HR, 0.91 [95% CI, 0.82-1.02]) (Table 2).

**Table 2.** Outcome Events Associated With Sacubitril-Valsartan Use in Medicare Recipients With HFrEF on Hemodialysis

Outcome	No. of events by treatment		IR (95% CI) per 1000 person-years by treatment		IRD (95% CI)	HR (95% CI)
	Sacubitril-valsartan	ACEI or ARB	Sacubitril-valsartan	ACEI or ARB		
**Intention to treat**						
All-cause mortality	554	618	318 (293 to 346)	391 (361 to 423)	−74 (−110 to −35)	0.82 (0.73 to 0.92)
CV mortality	299	272	172 (153 to 192)	172 (153 to 194)	0 (−26 to 29)	1.01 (0.86 to 1.19)
Any hospitalization	1057	1083	1379 (1297 to 1464)	1694 (1595 to 1797)	−322 (−424 to −186)	0.86 (0.79 to 0.93)
HF hospitalization	631	649	653 (604 to 706)	748 (692 to 807)	−97 (−165 to −22)	0.91 (0.82 to 1.02)
**As treated**						
All-cause mortality	232	286	242 (212 to 274)	317 (281 to 355)	−76 (−114 to −29)	0.80 (0.67 to 0.95)
CV mortality	132	135	138 (115 to 162)	149 (126 to 176)	−12 (−42 to 25)	0.97 (0.76 to 1.23)
Any hospitalization	808	889	1518 (1415 to 1625)	1927 (1803 to 2056)	−405 (−539 to −250)	0.84 (0.77 to 0.93)
HF hospitalization	488	539	771 (705 to 842)	939 (861 to 1020)	−169 (−253 to −66)	0.89 (0.79 to 1.01)

Abbreviations: ACEI, angiotensin-converting enzyme inhibitor; ARB, angiotensin receptor blocker; CV, cardiovascular; HF, heart failure; HR, hazard ratio; IR, incidence rate; IRD, incidence rate difference.

In the AT analysis, censoring due to medication discontinuation was high, with 809 patients (56%) stopping sacubitril-valsartan therapy after a median of 85 (IQR, 60-214) days, and 787 (55%) stopped ACEI or ARB therapy after 120 (IQR, 60-278) days, with 330 (23%) receiving sacubitril-valsartan for at least 1 year and 288 (20%) receiving ACEIs or ARBs. There were 232 deaths (16%) among participants receiving sacubitril-valsartan and 286 (20%) receiving ACEIs or ARBs with estimates similar to ITT analyses (Table 2).

Results of subgroup analysis for all-cause mortality and all-cause hospitalization in both ITT and AT analyses were generally consistent across age, sex, race, smoking status, dysrhythmia, atherosclerotic heart disease, diabetic kidney disease, history of diabetes, and diuretic use (eFigures 3 and 4 in Supplement 1) with some significant interaction effects. None, however, were consistent across both all-cause mortality and all-cause hospitalization. Similarly, we did not observe increased benefit in those with lower dialysis vintage. Instead, we observed that patients with higher dialysis vintage tended toward more benefit, though they were younger and healthier compared with those with lower dialysis vintage (eTable 5 in Supplement 1).

For safety outcomes, sacubitril-valsartan therapy was associated with a lower risk of hyperkalemia (HR, 0.71 [95% CI, 0.62-0.81]) and similar risk of hypotension (HR, 0.99 [95% CI, 0.83-1.19]) (Table 3). As-treated estimates were similar.

**Table 3.** Hyperkalemia and Hypotension Associated With Sacubitril-Valsartan Use in Medicare Recipients With HFrEF Receiving Hemodialysis

Safety outcome^a^	No. of events by treatment		IR (95% CI) per 1000 person-years by treatment		IRD (95% CI)	HR (95% CI)
	Sacubitril-valsartan	ACEI or ARB	Sacubitril-valsartan	ACEI or ARB		
**Intention to treat**						
Hyperkalemia (any)	367	456	276 (249 to 305)	414 (377 to 453)	−136 (−174 to −95)	0.71 (0.62 to 0.81)
Hyperkalemia (hospitalization claim)	295	381	214 (190 to 239)	331 (299 to 365)	−116 (−149 to −83)	0.69 (0.59 to 0.80)
Hypotension (any)	246	232	163 (143 to 184)	169 (148 to 191)	−5 (−32 to 27)	0.99 (0.83 to 1.19)
Hypotension (hospitalization claim)	11	<10^b^	6 (3 to 11)	5 (2 to 9)	1 (−3 to 11)	1.30 (0.52 to 3.23)
**As treated**						
Hyperkalemia (any)	241	338	299 (263 to 339)	482 (433 to 536)	−183 (−227 to −130)	0.67 (0.57 to 0.80)
Hyperkalemia (hospitalization claim)	201	286	242 (210 to 278)	396 (352 to 444)	−155 (−194 to −107)	0.67 (0.56 to 0.80)
Hypotension (any)	131	149	149 (125 to 176)	177 (150 to 207)	−28 (−58 to 12)	0.90 (0.71 to 1.13)
Hypotension (hospitalization claim)	<10^b^	<10^b^	4 (1 to 10)	7 (3 to 13)	−2 (−5 to 8)	0.67 (0.19 to 2.38)

Abbreviations: ACEI, angiotensin-converting enzyme inhibitor; ARB, angiotensin receptor blocker; HFrEF, heart failure with reduced ejection fraction; HR, hazard ratio; IR, incidence rate; IRD, incidence rate difference.

^a^ Defined by codes from *International Classification of Diseases, Ninth Revision,* and *International Statistical Classification of Diseases, Tenth Revision*.

^b^ Cells with less than 10 participants were censored per US Renal Data System reporting policy.

Of 1434 sacubitril-valsartan users, 70 (5%) started maximum dose of sacubitril-valsartan (97 mg twice a day [BID] and 103 mg BID, respectively); 249 (17%) started at 49 mg and BID and 51 mg BID, respectively; 1115 (78%) started at the lowest dose (24 mg BID and 26 mg BID, respectively). Compared with those receiving the lowest dose, individuals starting with the maximum dose were younger, less likely to have a recent hospitalization, and were generally healthier (eTable 6 in Supplement 1). When we examined for a dose-response relationship with starting dose among new users of sacubitril-valsartan, there was no association with all-cause mortality or hospitalization (eFigure 4 in Supplement 1). Titration was low with only 195 participants (14%) receiving the maximum dose and 900 (63%) receiving the lowest dose (eFigure 5 in Supplement 1). For comparison, 365 ACEI or ARB users (25%) received a maximum dose.

### Sensitivity Analysis

Results with 1:4 matching were consistent with 1:1 matching, and results with a 60-day (instead of 30-day) refill window were similar. There was no difference in fracture hospitalization in ITT (HR, 0.96 [95% CI, 0.76-1.21]) or AT (HR, 0.89 [95% CI, 0.67-1.18]) analysis. The E-value for mortality was 1.74, and the highest adjusted odds ratios (OR) for major study risk factors and all-cause mortality (n = 31 088) were current smoking status (OR 1.25) and diabetes (OR 1.15). Finally, estimates from competing risk models were similar (eTable 7 in Supplement 1).

## Discussion

In this active comparator, 1:1 propensity score–matched cohort of US patients receiving hemodialysis from 2015 to 2020, initiation of sacubitril-valsartan therapy among patients with HFrEF was associated with decreased all-cause mortality and all-cause hospitalization (compared with ACEI or ARB initiation or continued use). There was no association with CV mortality or HF hospitalization, and there were no consistent subgroup associations for both all-cause mortality and all-cause hospitalization. Sacubitril-valsartan was associated with a decreased risk of hyperkalemia and a similar risk of hypotension. There was a high rate of discontinuation of both sacubitril-valsartan therapy (56%) and ACEI or ARB therapy (55%), but AT point estimates were consistent with ITT analyses. Taken together, our results demonstrate that sacubitril-valsartan may have beneficial effects in all-cause mortality and hospitalization among patients with HFrEF requiring hemodialysis.

Our findings expand on studies in Asian patients with HF requiring dialysis^14,15,16,17,18,19^ where sacubitril-valsartan therapy was associated with biochemical (levels of high-sensitivity troponin T, BNP, and NT-proBNP) and echocardiographic improvement suggestive of clinical benefit. Given that Asian patients receiving hemodialysis have higher renal reserve (mean urine output of 500 [IQR, 100-1000] mL daily)^16,33^ and may benefit more from the diuretic effects of sacubitril,^34^ we looked at a subgroup interaction by diuretic use (as a proxy for residual renal function), which was negative. Only 2 prior observational studies^12,13^ have examined sacubitril-valsartan and clinical outcomes in patients receiving dialysis, and both studies were conducted in Asia. These studies, however, included patients with HF with preserved EF^12^ as well as those not requiring dialysis.^13^ Both studies did not observe a mortality benefit, but power was limited with a combined study population receiving sacubitril-valsartan of less than 200 (vs 1434 for this study and 4187 in PARADIGM-HF).^13,35^ Additionally, one of these studies compared sacubitril-valsartan use vs no medication use, raising concern for bias (ie, confounding by indication).^12,36^ Importantly, we saw a beneficial association despite the high morbidity and mortality among patients receiving dialysis, with 1-year mortality at 30% vs 10% to 15% in Asia^12,13^ (and <10% in PARADIGM-HF).^2^ We additionally examined dialysis vintage as those newly receiving dialysis may have less cardiac remodeling, thereby allowing more benefit.^37^ There was no significant interaction, but paradoxically, those with higher dialysis vintage tended toward more benefit, potentially due to confounding from younger age and less comorbidity.

We did not observe a significant difference in CV mortality or HF hospitalization, which contrasts with the PARADIGM-HF trial. Importantly, our cause-specific outcomes (HF hospitalization [defined by *ICD-9* and *ICD-10* codes] and CV mortality [defined via the USRDS]) may be susceptible to misclassification. Sacubitril-valsartan (vs ACEI or ARB) users may have different *ICD* billing practices (such as increased number of *ICD-9* or *ICD-10* billed codes),^6^ and this may help explain the inconsistent association with HF hospitalization among individuals requiring dialysis (ie, increased,^13^ decreased,^12^ or neutral [in this study]). Similarly, cause of death in the USRDS has been shown to have only moderate agreement with an integrated health system.^38^ We saw reductions in all-cause mortality and all-cause hospitalization, which is immune to this type of misclassification. Conversely, we may have lacked power for cause-specific outcomes (ie, HF hospitalization HR, 0.91 [95% CI, 0.82-1.02]). Further studies are needed to confirm our findings (like ongoing Asian clinical trials)^10,11^ and should consider prospective adjudication of outcomes similar to PARADIGM-HF.^2^

Alternatively, the reduction in all-cause mortality but not cardiovascular mortality with sacubitril-valsartan (vs ACEIs or ARBs) could be related to decreases in hyperkalemia,^39,40,41,42^ which is common in patients receiving dialysis. Previous studies have suggested that patient factors (and not dialysis prescription) have stronger associations with serum potassium levels and that potassium-specific interventions (like medications) could improve clinical outcomes.^39^ Considering that the interplay among potassium level,^43,44,45,46^ renin-angiotensin-aldosterone system inhibition (via ACEI, ARB, or ARNI), and clinical outcomes is complex,^47,48^ further investigation is warranted as it may contribute to our positive findings.

For hypotension, there was not a significant difference, but 63% of patients continued to receive the starting dose of sacubitril-valsartan (ie, 24 and 26 mg, BID, respectively). Additionally, the rate of hypotensive events was 10-fold higher than the general population^6^ and may have contributed to low dose titration. Given that less benefit for sacubitril-valsartan has been seen in individuals with lower blood pressures,^49,50,51^ we examined the association between the starting dose and clinical outcomes among sacubitril-valsartan new users, which was negative. This observation is similar to those of other studies that specifically examined sacubitril-valsartan doses and clinical outcomes.^52,53^

### Strengths and Limitations

Strengths of this study include the active comparator design, complete outcome ascertainment due to Medicare and USRDS, the largest sample, to our knowledge, of patients with HFrEF undergoing hemodialysis to date, and the first non-Asian observational data. Our study also has limitations. First, we included participants with prevalent use of ACEs or ARBs, which can introduce selection bias (as individuals with prevalent ACEI or ARB use already tolerate therapy and may be “healthier” compared with those with incident ACEI or ARB use). If this bias was present after propensity score matching, however, it would bias our results toward the null and not explain our positive finding.^54^ Next, we were unable to account for residual or unmeasured confounding seen in observational studies (ie, New York Heart Association class or echocardiographic measurements). Using the E-value method, an unmeasured confounder would need to be associated with both sacubitril-valsartan and all-cause mortality by a risk ratio of at least 1.74 to explain our observed benefit. Within our eligible patients, the highest OR of mortality and major risk factors was less than 1.3 (ie, smoking OR, 1.25). Therefore, the possibility of an unknown confounder having an association of greater magnitude is low. There could also be confounding by indication where healthier patients received sacubitril-valsartan despite our propensity score match and negative control outcome. We used medication dispensing history, which is better than prescription data, but misclassification risk remains. Due to limited power, we were unable to explore sacubitril-valsartan titration and outcomes. Individuals also had at least 180 days of Medicare primary payer status, which limits generalizability. Finally, multiple covariates and outcomes (like HFrEF and hypotension) were defined by *ICD* codes that are susceptible to measurement error.

## Conclusions

In this comparative effectiveness study with a 1:1 matched cohort of US Medicare beneficiaries in the USRDS registry from 2015 to 2020 with HFrEF requiring hemodialysis, initiation of sacubitril-valsartan (vs ACEI or ARB) therapy was associated with improved all-cause mortality and hospitalization but not with improved cardiovascular mortality or HF hospitalization. Therapy was well tolerated without an increase in hyperkalemia or hypotension, and AT effect estimates were similar despite at least 50% of patients discontinuing therapy during follow-up. Our study shows that sacubitril-valsartan may have significant therapeutic potential among patients with HFrEF requiring hemodialysis, but further studies are needed before changes in clinical practice.

## Notes

Supplement 1. **eTable 1.** Specification and Emulation of a Target Trial for Sacubitril-Valsartan vs ACEI or ARB in US Medicare Patients **eTable 2.** Specific Medications Assessed Using Medicare Part D Dispense Records **eTable 3.** *ICD-9*, *ICD-10*, and *CPT* Codes for Comorbidity and Outcome Assessment **eTable 4.** ACEI or ARB Stratification by Total Daily Dose **eTable 5.** Baseline Characteristics of Sacubitril-Valsartan Users by Quartile of Dialysis Vintage **eTable 6.** Baseline Characteristics of Sacubitril-Valsartan Users by Initial Dispensed Dose **eTable 7.** HR of All-Cause Mortality, CV Mortality, Any Hospitalization, HF Hospitalization, Hyperkalemia, and Hypotension Associated With Sacubitril-Valsartan Use (vs ACEI or ARB) in Medicare Recipients With HFrEF Receiving Hemodialysis **eFigure 1.** Covariate Balance and Post 1:1 Propensity Score Matching Without Replacement **eFigure 2.** Subgroup Analyses of the HR for All-Cause Mortality Comparing Sacubitril-Valsartan vs ACEI or ARB After 1:1 Match Across Demographics, Medical Comorbidities, and Medication Dispensed History **eFigure 3.** Subgroup Analyses of the HR for All-Cause Hospitalization Comparing Sacubitril-Valsartan vs ACEI or ARB **eFigure 4.** Dose-Response Relationship Comparing Both All-Cause Mortality and All-Cause Hospitalization With Sacubitril-Valsartan Initiation Dose **eFigure 5.** Sacubitril/Valsartan Dosing Pattern During Follow-Up Defined Using the Starting Dispenses Dose, Maximum Dispensed Dose, and Most Recent Dispensed Dose

Supplement 2.
Data Sharing Statement
