# Supplementary material for: Sacubitril-Valsartan in Patients Requiring Hemodialysis
Source: JAMA Netw Open. 2024 Aug 20;7(8):e2429237. doi: 10.1001/jamanetworkopen.2024.29237 (PMC11337068; doi:10.1001/jamanetworkopen.2024.29237)
Supplement: Supplement 1. — eTable 1. Specification and Emulation of a Target Trial for Sacubitril-Valsartan vs ACEI or ARB in US Medicare Patients eTable 2. Specific Medications Assessed Using Medicare Part D Dispense Records eTable 3. ICD-9, ICD-10, and CPT Codes for Comorbidity and Outcome Assessment eTable 4. ACEI or ARB Stratification by Total Daily Dose eTable 5. Baseline Characteristics of Sacubitril-Valsartan Users by Quartile of Dialysis Vintage eTable 6. Baseline Characteristics of Sacubitril-Valsartan Users by Initial Dispensed Dose eTable 7. HR of All-Cause Mortality, CV Mortality, Any Hospitalization, HF Hospitalization, Hyperkalemia, and Hypotension Associated With Sacubitril-Valsartan Use (vs ACEI or ARB) in Medicare Recipients With HFrEF Receiving Hemodialysis eFigure 1. Covariate Balance and Post 1:1 Propensity Score Matching Without Replacement eFigure 2. Subgroup Analyses of the HR for All-Cause Mortality Comparing Sacubitril-Valsartan vs ACEI or ARB After 1:1 Match Across Demographics, Medical Comorbidities, and Medication Dispensed History eFigure 3. Subgroup Analyses of the HR for All-Cause Hospitalization Comparing Sacubitril-Valsartan vs ACEI or ARB eFigure 4. Dose-Response Relationship Comparing Both All-Cause Mortality and All-Cause Hospitalization With Sacubitril-Valsartan Initiation Dose eFigure 5. Sacubitril/Valsartan Dosing Pattern During Follow-Up Defined Using the Starting Dispenses Dose, Maximum Dispensed Dose, and Most Recent Dispensed Dose [file jamanetwopen-e2429237-s001.pdf]

# Supplementary Online Content

Le D, Grams M, Coresh J, Shin J-I. Combined sacubitril and valsartan in patients requiring hemodialysis. *JAMA Netw Open*. 2024;7(8):e2429237.
doi:10.1001/jamanetworkopen.2024.29237

**eTable 1.** Specification and Emulation of a Target Trial for Sacubitril-Valsartan vs ACEI/ARB in US Medicare Patients

**eTable 2.** Specific Medications Assessed Using Medicare Part D Dispense Records

**eTable 3.** *ICD-9*, *ICD-10*, and *CPT* Codes for Comorbidity and Outcome Assessment

**eTable 4.** ACEI/ARB Stratification by Total Daily Dose

**eTable 5.** Baseline Characteristics of Sacubitril-Valsartan Users by Quartile of Dialysis Vintage

**eTable 6.** Baseline Characteristics of Sacubitril-Valsartan Users by Initial Dispensed Dose

**eTable 7.** HR of All-Cause Mortality, CV Mortality, Any Hospitalization, HF Hospitalization, Hyperkalemia, and Hypotension Associated With Sacubitril-Valsartan Use (vs ACEI/ARB) in Medicare Recipients With HFrEF Receiving Hemodialysis

**eFigure 1.** Covariate Balance and Post 1:1 Propensity Score Matching Without Replacement

**eFigure 2.** Subgroup Analyses of the HR for All-Cause Mortality Comparing Sacubitril-Valsartan vs ACEI/ARB After 1:1 Match Across Demographics, Medical Comorbidities, and Medication Dispensed History

**eFigure 3.** Subgroup Analyses of the HR for All-Cause Hospitalization Comparing Sacubitril-Valsartan vs ACEI/ARB

**eFigure 4.** Dose-Response Relationship Comparing Both All-Cause Mortality and All-Cause Hospitalization With Sacubitril-Valsartan Initiation Dose

**eFigure 5.** Sacubitril/Valsartan Dosing Pattern During Follow-Up Defined Using the Starting Dispenses Dose, Maximum Dispensed Dose, and Most Recent Dispensed Dose

This supplementary material has been provided by the authors to give readers additional information about their work.

© 2024 Le D et al. *JAMA Network Open*.

**eTable 1.** Specification and Emulation of a Target Trial for Sacubitril-Valsartan vs ACEI/ARB in US Medicare Patients

| | Target Trial | Emulated Trial Using Real-World Data |
|---|---|---|
| Eligibility | Individuals ≥18 years old starting sacubitril/valsartan or ACE/ARB on-center hemodialysis between 2015 – 2020 with HFrEF | Same + ≥90-day survival on hemodialysis (given recommendations by the USRDS for observational data) + ≥180 days of Medicare A, B, and D (primary payer) prior to study initiation + history of HFrEF defined by ICD9 428.2X or ICD10 I50.2X |
| Treatment Strategies | Sacubitril/valsartan versus ACE/ARB | Same. Date of sacubitril/valsartan or ACE/ARB dispense was defined as study start |
| Treatment Assignmen t | Patients are assigned at random stratified by history of ACE/ARB use | Individuals were defined by treatment strategy at baseline, and we emulated randomization with 1:1 matching |
| Primary Outcome | All-cause mortality + cardiovascular death, HF hospitalization, all hospitalization | Same as for target trial (via ICD-9/ICD-10 codes) |
| Follow-up | Follow-up starts day after medication dispense and ends at study outcome, death, transplant, renal recovery, or loss of Medicare | Same as for target trial |
| Causal Contrast | Intention-to-treat | Observational analog of intention-to-treat effect |
| Statistical Analysis | Cox proportional hazards | Propensity score match with Cox proportional hazards |

Abbreviations: US, United States, USRDS, United States Renal Data System; ACE/ARB, Angiotensin Converting Enzyme inhibitor/Angiotensin Receptor Blocker; HFrEF, heart failure with reduced ejection fraction; ICD, International Classification of Disease; Cox PH, Cox proportional hazards; IPTW, inverse probability of treatment weighting.

© 2024 Le D et al. *JAMA Network Open*.

**eTable 2.** Specific Medications Assessed Using Medicare Part D Dispense Records

| Medication Class | Specific Medications |
|---|---|
| ACE/ARB | Azilsartan, Benazepril, Candesartan, Captopril, Enalapril, Fosinopril, Irbesartan, Lisinopril, Losartan, Olmesartan, Quinapril, Ramipril, Telmisartan, Valsartan |
| Aldosterone Antagonists | Eplerenone, Spironolactone |
| Antiplatelet | Aspirin, Cangrelor, Clopidogrel, Prasugrel, Ticagrelor, Ticlopidine |
| Beta Blockers | Atenolol, Bisoprolol, Carvedilol, Metoprolol, Nebivolol, Pindolol, Propranolol |
| CCB | Amlodipine, Diltiazem, Felodipine, Israldipine, Nicardipine, Nifedipine, Nimodipine, Nisoldipine, Verapamil |
| Diuretics | Chlorothiazide, Chlorthalidone, Hydrochlorothiazide, Indapamide, Metolazone, Bumetanide, Ethacrynic acid, Furosemide, Torsemide, Amiloride, Triamterene |
| DOAC | Apixaban, Dabigatran, Edoxaban, Rivarxoaban |
| GLP1 Agonists | Albiglutide, Dulaglutide, Exenatide, Liraglutide, Lixisenatide, Semaglutide, Tirzepatide |
| Insulin | Insulin |
| Phosphate Binders | Sevelamer Carbonate, Sevelamer Hydrochloride, Calcium Acetate, Ferric Citrate, Lanthanum Carbonate, Sucroferric Oxyhydroxide, Calcium Citrate |
| SGLT2i | Dapagliflozin, Canagliflozin, Empagliflozin, Ertugliflozin |
| Warfarin | Warfarin |

Abbreviations: ACE, angiotensin converting enzyme; ARB, angiotensin receptor blockers; CCB, calcium channel blockers; DOAC, Direct Oral Anticoagulation; GLP-1, Glucagon Like Peptide – 1 Agonists; SGLT2i, Sodium-glucose cotransporter-2 inhibitor.

© 2024 Le D et al. *JAMA Network Open*.

eTable 3. *ICD-9*, *ICD-10*, and *CPT* Codes for Comorbidity and Outcome Assessment

| Comorbidities | ICD9-CM Codes | ICD10-CM Codes |
|---|---|---|
| Heart Failure with Reduced Ejection Fraction | 428.2X | I50.2X |
| | | |
| Atherosclerotic Heart Disease | 410 – 414; V45.81, V45.82 | I21, I22, I25; Z95, Z9861 |
| Cerebrovascular Disease | 430 – 438 | G45, G46, H340, H341, H341, I60 – I69 |
| Peripheral Vascular Disease | 440 – 444, 447, 451, 453, 557; | I70 – I74, I77, I791, I80, I731, I738, I739, I771, I790, K551, K558, K559 |
| Gastrointestinal Bleeding | 456.20, 530.21, 530.7, 530.82, 531.x-534.x (x=0,2,4,6), 535.x1 (x=1-7), 537.83, 537.84, 578.0, 562.02, 562.03, 562.12, 562.13, 569.3, 569.85,568.81, 569.86, 578.1, 578.9, 79.21 | I85.01, I85.11, K22.11, K22.6, K25, K26 K27, K28, K29.x1 (x=0-9), K31.81, K31.82, K57.13, K92.0, K92.1, K50.x11 (x=0,1,8,9), K51.x11 (x=0,2,3,4,5,8,9), K55.2, K57.x1 (x=0,1,2,3,8), K62.5, K57.33, K56.60, K92.2, K57.91, K57.93 |
| Other Cardiac Disease | 420 – 424, 429, 785 – 785.3; V42.2, V43.3 | 9309, I31 – I38, I47, I51, I52, R002, R011, R012; Z95.4, Z95.3, Z95.5 |
| Respiratory Disease | 491 – 494, 496, 510 | J01, J41, J43, J449, J45 – J47 |
| Liver Disease | 570, 571, 572.1, 572.4, 573.1 – 573.3; V42.7 | B18, K700 – K703, K709, K713 – K715, K72, K75.1, K67.7, K73, K74.60; Z94.4 |
| Dysrhythmia | 426, 427; V45.0, V53.3 | I44, I45, I47 – I49; Z95.0, Z95.810 |
| Cancer | 140 – 172, 174 – 208, 230 – 231, 233 – 234 | C0X – C34, C37 – C43, C45 – C58, C60 – C86, C88, C90 – C96 |
| Diabetes Mellitus | 250, 357.2, 362.0X, 366.1 | E08 – E11, E13 |
| | | |
| **Outcomes** | | |
| Heart Failure Hospitalization | 398.91, 422, 425, 428, 402.X1, 403.X1, 404.X1; V42.1 | I11, I13, I3.2, I2.55, I42.0, I42.5 – I42.9. I43.X, I40.9, I50, I09.81; Z94.1 |
| Hyperkalemia | 276.7 | E87.5 |
| Hypotension | 458.9 | I95.9 |
| Fracture | 733.1, 802, 804 – 808, 810 – 817, 820 – 829, E2887 | M484, M80, M84, Sx2 (x = 0 – 9) |

Abbreviations: ICD, International Classification of Disease; CM, Clinical Modification; GIB, gastrointestinal bleed; NOS, Not Otherwise Specified (Location of GIB); HCPCS, Healthcare Common Procedures Coding System; EGD, esophagogastroduodenoscopy.

© 2024 Le D et al. *JAMA Network Open*.

**eTable 4.** ACEI/ARB Stratification by Total Daily Dose

| | ACE/ARB Stratification by Total Daily Dose (mg) | | |
|---|---|---|---|
| **Medication** | **Low** | **Intermediate** | **High** |
| Azilsartan | - | 40 | >80 |
| Benazepril | ≤5 | >5 - ≤15 | >15 |
| Candesartan | ≤4 | >4 - ≤16 | >16 |
| Captopril | ≤37.5 | >37.5 - ≤75 | >75 |
| Enalapril | ≤5 | >5 - ≤20 | >20 |
| Fosinopril | ≤5 | >5 - ≤20 | >20 |
| Irbesartan | ≤75 | >75 - ≤150 | >150 |
| Lisinopril | ≤5 | >5 - ≤20 | >20 |
| Losartan | ≤25 | >25 - ≤75 | >75 |
| Olmesartan | ≤10 | >10 - ≤20 | >20 |
| Quinapril | 5 | 10 | >10 |
| Ramipril | ≤2.5 | >2.5 - ≤7.5 | >7.5 |
| Telmisartan | ≤20 | >20 - ≤40 | >40 |
| Valsartan | ≤40 | >40 - ≤160 | >160 |

Medication doses determined via National Drug Code (NDC) linkage with billed Medicare Part D dispense

© 2024 Le D et al. *JAMA Network Open*.

**eTable 5.** Baseline Characteristics of Sacubitril-Valsartan Users by Quartile of Dialysis Vintage

| | Dialysis Vintage in Months<br><22 Months<br>N = 743 | <br>22 – 44 Months<br>N = 703 | <br>45 – 74 Months<br>N = 721 | <br>>75 Months<br>N = 701 | p |
|---|---|---|---|---|---|
| Age (Years) | 66.2 (13.6) | 64.4 (14.4) | 63.7 (13.3) | 61.9 (12.6) | <0.01 |
| Sex (Male) | 488 ( 65.7) | 466 ( 66.3) | 468 ( 64.9) | 450 ( 64.2) | 0.86 |
| Race | | | | | <0.01 |
| White | 501 ( 67.4) | 462 ( 65.7) | 398 ( 55.2) | 316 ( 45.1) | |
| Black or AA | 189 ( 25.4) | 191 ( 27.2) | 277 ( 38.4) | 330 ( 47.1) | |
| Other | 53 ( 7.1) | 50 ( 7.1) | 46 ( 6.4) | 55 ( 7.8) | |
| Baseline Calendar Year | | | | | 0.12 |
| 2015 | <10* | <10* | <10* | <10* | |
| 2016 | 34 ( 4.6) | 40s* | 50s* | 30s* | |
| 2017 | 98 ( 13.2) | 79 ( 11.2) | 105 ( 14.6) | 85 ( 12.1) | |
| 2018 | 137 ( 18.4) | 129 ( 18.3) | 135 ( 18.7) | 145 ( 20.7) | |
| 2019 | 240 ( 32.3) | 231 ( 32.9) | 237 ( 32.9) | 219 ( 31.2) | |
| 2020 | 234 ( 31.5) | 217 ( 30.9) | 193 ( 26.8) | 222 ( 31.7) | |
| KT/V | 1.5 [1.4, 1.7] | 1.5 [1.4, 1.7] | 1.6 [1.4, 1.7] | 1.6 [1.4, 1.7] | 0.11 |
| Smoker at Dialysis Initiation | 64 ( 8.6) | 55 ( 7.8) | 46 ( 6.4) | 53 ( 7.6) | 0.45 |
| Medicare Part D Subsidy | 440 ( 59.2) | 475 ( 67.6) | 517 ( 71.7) | 566 ( 80.7) | <0.01 |
| History of ESA use | 514 ( 69.2) | 577 ( 82.1) | 658 ( 91.3) | 650 ( 92.7) | <0.01 |
| Kidney Failure Not from Diabetes | 365 ( 49.1) | 357 ( 50.8) | 347 ( 48.1) | 413 ( 58.9) | <0.01 |
| Geographic Region | | | | | <0.01 |
| Midwest | 180 ( 24.2) | 156 ( 22.2) | 144 ( 20.0) | 120 ( 17.1) | |
| Northeast | 187 ( 25.2) | 173 ( 24.6) | 201 ( 27.9) | 159 ( 22.7) | |
| South | 247 ( 33.2) | 243 ( 34.6) | 255 ( 35.4) | 302 ( 43.1) | |
| West | 129 ( 17.4) | 131 ( 18.6) | 121 ( 16.8) | 120 ( 17.1) | |
| Started Dialysis Via Catheter | 639 (86.0) | 576 (81.9) | 552 (76.6) | 476 (67.9) | <0.01 |
| BMI (kg/m$^2$) | 26.0 [22.4, 30.3] | 26.7 [22.8, 31.6] | 26.7 [23.0, 31.2] | 26.0 [22.4, 30.2] | 0.07 |
| Atherosclerotic Heart Disease | 452 ( 60.8) | 395 ( 56.2) | 424 ( 58.8) | 356 ( 50.8) | <0.01 |
| Cancer | 95 ( 12.8) | 89 ( 12.7) | 73 ( 10.1) | 72 ( 10.3) | 0.21 |
| Dysrhythmia | 403 ( 54.2) | 321 ( 45.7) | 341 ( 47.3) | 320 ( 45.6) | <0.01 |
| Cerebrovascular Disease | 158 ( 21.3) | 145 ( 20.6) | 173 ( 24.0) | 135 ( 19.3) | 0.17 |
| Other Cardiac Disease | 411 ( 55.3) | 387 ( 55.0) | 411 ( 57.0) | 347 ( 49.5) | 0.03 |
| Diabetes | 565 ( 76.0) | 519 ( 73.8) | 525 ( 72.8) | 435 ( 62.1) | <0.01 |
| History of GI Bleed | 86 ( 11.6) | 71 ( 10.1) | 75 ( 10.4) | 86 ( 12.3) | 0.53 |
| Hypertension | 647 ( 87.1) | 633 ( 90.0) | 651 ( 90.3) | 605 ( 86.3) | 0.03 |
| Inability to Ambulate | 32 ( 4.3) | 29 ( 4.1) | 20 ( 2.8) | <10* | <0.01 |
| Liver Disease | 86 ( 11.6) | 75 ( 10.7) | 81 ( 11.2) | 80 ( 11.4) | 0.95 |
| Peripheral Vascular Disease | 393 ( 52.9) | 348 ( 49.5) | 344 ( 47.7) | 350 ( 49.9) | 0.25 |
| Respiratory Disease | 215 ( 28.9) | 187 ( 26.6) | 191 ( 26.5) | 153 ( 21.8) | 0.02 |
| # of Hospitalizations [≤ 6 mo] | 2.0 (2.0) | 1.8 (1.9) | 1.8 (1.8) | 1.6 (1.8) | <0.01 |

© 2024 Le D et al. *JAMA Network Open*.

| | | | | | |
|---|---|---|---|---|---|
| # of PCP Visits [≤ 6 mo] | 2.4 (3.5) | 2.3 (3.3) | 2.1 (3.2) | 2.4 (3.5) | 0.32 |
| # of Medications [<90 days] | 10.0 [7.0, 14.0] | 10.0 [7.0, 14.0] | 10.0 [7.0, 14.0] | 10.0 [7.0, 13.0] | 0.13 |
| Aldosterone Antagonist | 59 ( 7.9) | 30 ( 4.3) | 44 ( 6.1) | 31 ( 4.4) | 0.01 |
| Antiplatelets | 218 ( 29.3) | 234 ( 33.3) | 215 ( 29.8) | 180 ( 25.7) | 0.02 |
| Beta-Blockers | 584 ( 78.6) | 549 ( 78.1) | 554 ( 76.8) | 541 ( 77.2) | 0.84 |
| Calcium Channel Blockers | 194 ( 26.1) | 209 ( 29.7) | 214 ( 29.7) | 206 ( 29.4) | 0.35 |
| Diuretics | 310 ( 41.7) | 218 ( 31.0) | 177 ( 24.5) | 93 ( 13.3) | <0.01 |
| DOAC | 103 ( 13.9) | 95 ( 13.5) | 74 ( 10.3) | 110 ( 15.7) | 0.02 |
| GLP-1RA | 11 ( 1.5) | <10* | <10* | <10* | 0.3 |
| Insulin | 208 ( 28.0) | 187 ( 26.6) | 194 ( 26.9) | 114 ( 16.3) | <0.01 |
| Phosphate Binder | 371 ( 49.9) | 414 ( 58.9) | 427 ( 59.2) | 430 ( 61.3) | <0.01 |
| SGLT2i | <10* | <10* | <10* | <10* | 0.58 |
| Warfarin | 82 ( 11.0) | 62 ( 8.8) | 60 ( 8.3) | 65 ( 9.3) | 0.31 |
| Baseline ACE/ARB use | | | | | <0.01 |
| Low Dose | 129 ( 17.4) | 120 ( 17.1) | 107 ( 14.8) | 111 ( 15.8) | |
| Moderate Dose | 122 ( 16.4) | 133 ( 18.9) | 145 ( 20.1) | 141 ( 20.1) | |
| High Dose | 71 ( 9.6) | 94 ( 13.4) | 118 ( 16.4) | 101 ( 14.4) | |
| No use | 421 ( 56.7) | 356 ( 50.6) | 351 ( 48.7) | 348 ( 49.6) | |
| # of ACE/ARB Dispenses [≤ 6 mo] | 1.1 (1.8) | 1.3 (1.8) | 1.4 (2.1) | 1.3 (1.9) | 0.05 |

Data reported as number (percent), mean (standard deviation)
*Cells with <10 were censored per USRDS reporting policy or rounded down (if multiple categories).
Abbreviations: SMD, absolute standardized mean difference; AA, African American, ESA, erythropoietin stimulating agent; BMI, body mass index; ACEARB, angiotensin converting enzyme/angiotensin receptor blocker; CCB, calcium-channel blocker; DOAC; KT/V, measure of dialysis adequacy; Direct oral anticoagulation; GLP, Glucagon-Like-Peptide 1; SGLT2, sodium-glucose cotransporter 2 inhibitor

© 2024 Le D et al. *JAMA Network Open*.

**eTable 6.** Baseline Characteristics of Sacubitril-Valsartan Users by Initial Dispensed Dose

| | Starting Dispense of Sacubitril/Valsartan | | | |
|---|---|---|---|---|
| | 24/26 mg<br>N = 1115 | 49/51 mg<br>N = 249 | 97/101 mg<br>N = 70 | P-value |
| Age | 65.3 (12.7) | 61.3 (14.0) | 61.0 (15.1) | <0.01 |
| Sex (Male) | 734 (65.8) | 157 (63.1) | 53 (75.7) | 0.14 |
| Race | | | | 0.32 |
| White | 669 (60.0) | 133 (53.4) | 38 (54.3) | |
| Black or AA | 373 (33.5) | 100 (40.2) | 20s* | |
| Other | 73 ( 6.5) | 16 ( 6.4) | <10* | |
| Year | | | | 0.61 |
| 2015 | <10* | <10* | <10* | |
| 2016 | 60s* | 10s* | <10* | |
| 2017 | 129 (11.6) | 37 (14.9) | 13 (18.6) | |
| 2018 | 209 (18.7) | 48 (19.3) | 13 (18.6) | |
| 2019 | 374 (33.5) | 70 (28.1) | 20 (28.6) | |
| 2020 | 337 (30.2) | 77 (30.9) | 19 (27.1) | |
| Dialysis Vintage | 3.8 (1.8 – 6.3) | 3.9 (2.1 – 6.1) | 4.3 (1.8 – 6.0) | 0.90 |
| KT/V | 1.5 [1.4, 1.7] | 1.5 [1.4, 1.7] | 1.5 [1.4, 1.7] | 0.66 |
| Current Smoker | 78 ( 7.0) | 25 (10.0) | 7 (10.0) | 0.20 |
| Part D Subsidy | 755 (67.7) | 177 (71.1) | 45 (64.3) | 0.46 |
| History of ESA Use | 926 (83.0) | 213 (85.5) | 62 (88.6) | 0.34 |
| Non-Diabetic Kidney Failure | 569 (51.0) | 143 (57.4) | 39 (55.7) | 0.16 |
| US Geographic Region | | | | 0.74 |
| Midwest | 232 (20.8) | 51 (20.5) | 19 (27.1) | |
| Northeast | 291 (26.1) | 59 (23.7) | 14 (20.0) | |
| South | 400 (35.9) | 98 (39.4) | 24 (34.3) | |
| West | 192 (17.2) | 41 (16.5) | 13 (18.6) | |
| Started Dialysis Via Catheter | 975 (80%) | 209 (77%) | 57 (77%) | 0.69 |
| BMI | 26.2 [22.5, 30.4] | 25.0 [22.6, 29.7] | 27.3 [24.0, 30.9] | 0.13 |
| Atherosclerotic Heart Disease | 668 (59.9) | 117 (47.0) | 40 (57.1) | <0.01 |
| Cancer | 127 (11.4) | 25 (10.0) | <10* | 0.48 |
| Dysrhythmia | 580 (52.0) | 92 (36.9) | 28 (40.0) | <0.01 |
| Cerebrovascular Disease | 245 (22.0) | 49 (19.7) | 14 (20.0) | 0.69 |
| Other Cardiac Disease | 612 (54.9) | 131 (52.6) | 43 (61.4) | 0.42 |
| Diabetes | 809 (72.6) | 166 (66.7) | 48 (68.6) | 0.16 |
| Hx of Gastrointestinal Bleed | 142 (12.7) | 23 ( 9.2) | <10* | 0.21 |
| Hypertension | 974 (87.4) | 225 (90.4) | 64 (91.4) | 0.28 |
| Inability to Ambulate | 35 ( 3.1) | <10* | <10* | 0.22 |
| Liver Disease | 123 (11.0) | 30 (12.0) | 8 (11.4) | 0.90 |
| Peripheral Vascular Disease | 589 (52.8) | 105 (42.2) | 23 (32.9) | <0.01 |

© 2024 Le D et al. *JAMA Network Open*.

| | | | | |
|---|---|---|---|---|
| Respiratory Disease | 295 (26.5) | 61 (24.5) | 14 (20.0) | 0.43 |
| # of Hospitalizations [≤ 6 mo] | 1.8 (1.9) | 1.6 (1.9) | 1.7 (2.7) | 0.08 |
| # of PCP Visits [≤ 6 mo] | 2.2 (3.0) | 2.1 (2.9) | 2.9 (5.2) | 0.14 |
| # of Medications [< 90 days] | 10.0 [7.0, 14.0] | 10.0 [7.0, 14.0] | 9.0 [6.0, 13.0] | 0.14 |
| Aldosterone Antagonist | 54 ( 4.8) | 20 ( 8.0) | <10* | 0.02 |
| Antiplatelets | 358 (32.1) | 54 (21.7) | 18 (25.7) | <0.01 |
| Beta-Blockers | 880 (78.9) | 192 (77.1) | 52 (74.3) | 0.57 |
| CCB | 297 (26.6) | 85 (34.1) | 19 (27.1) | 0.06 |
| Diuretics | 304 (27.3) | 71 (28.5) | 21 (30.0) | 0.83 |
| DOAC | 154 (13.8) | 24 ( 9.6) | <10* | 0.11 |
| GLP-1RA | 12 ( 1.1) | <10* | <10* | 0.64 |
| Insulin | 287 (25.7) | 54 (21.7) | <10* | 0.05 |
| Phosphate Binder | 629 (56.4) | 136 (54.6) | 31 (44.3) | 0.13 |
| SLGT2 | <10* | <10* | <10* | 0.87 |
| Warfarin | 106 ( 9.5) | 21 ( 8.4) | <10* | 0.72 |
| Baseline ACE/ARB use | | | | <0.01 |
| Low Dose | 194 (17.4) | 31 (12.4) | <10* | |
| Moderate Dose | 186 (16.7) | 68 (27.3) | 10s* | |
| High Dose | 114 (10.2) | 56 (22.5) | 19 (27.1) | |
| No use | 621 (55.7) | 94 (37.8) | 34 (48.6) | |
| # of ACE/ARB Refills [≤ 6 mo] | 1.2 (1.8) | 1.7 (2.0) | 1.5 (1.8) | <0.01 |

Abbreviations: SMD, absolute standardized mean difference; AA, African American, ESA, erythropoietin stimulating agent; BMI, body mass index; GI, gastrointestinal; mo, months; #, Number; DOAC, Direct Oral Anticoagulation; GLP-1RA, Glucagon Like Peptide – 1 Receptor Agonists; SGLT2, Sodium-glucose cotransporter-2 inhibitor; ACE/ARB, angiotensin converting enzyme/angiotensin receptor blocker
*Cells with <10 were censored per USRDS reporting policy or rounded down (if multiple categories).

© 2024 Le D et al. *JAMA Network Open*.

**eTable 7.** HR of All-Cause Mortality, CV Mortality, Any Hospitalization, HF Hospitalization, Hyperkalemia, and Hypotension Associated With Sacubitril-Valsartan Use (vs ACEI/ARB) in Medicare Recipients With HFrEF Receiving Hemodialysis

| | No. of Events | | IR (95% CI), per 1000 PYs | | | |
|---|---|---|---|---|---|---|
| ***Intention To Treat*** | **Sacubitril/ valsartan** | **ACE/ ARB** | **Sacubitril/ valsartan** | **ACE/ARB** | **HR** | **CRR*** |
| All-Cause Mortality | 554 | 618 | 318 (293, 346) | 391 (361, 423) | 0.82 (0.73 - 0.92) | |
| CV Mortality | 299 | 272 | 172 (153, 192) | 172 (153, 194) | 1.01 (0.86 - 1.19) | 1.09 (0.92 – 1.28) |
| Any Hospitalization | 1,057 | 1,083 | 1,379 (1,297, 1464) | 1,694 (1,595, 1,797) | 0.86 (0.79 - 0.93) | 0.88 (0.81 – 0.96) |
| HF hospitalization | 631 | 649 | 653 (604, 706) | 748 (692, 807) | 0.91 (0.82 - 1.02) | 0.93 (0.84 – 1.04) |
| **As Treated** | **Sacubitril/ valsartan** | **ACE/ ARB** | **Sacubitril/ valsartan** | **ACE/ARB** | **HR** | **CRR** |
| All-Cause Mortality | 232 | 286 | 242 (212, 274) | 317 (281, 355) | 0.80 (0.67 - 0.95) | |
| CV Mortality | 132 | 135 | 138 (115, 162) | 149 (126, 176) | 0.97 (0.76 - 1.23) | 1.01 (0.80 – 1.29) |
| Any Hospitalization | 808 | 889 | 1,518 (1415, 1625) | 1,927 (1803, 2056) | 0.84 (0.77 - 0.93) | 0.87 (0.79 – 0.95) |
| HF hospitalization | 488 | 539 | 771 (705, 842) | 939 (861, 1020) | 0.89 (0.79 - 1.01) | 0.96 (0.86 – 1.07) |
| ***Intention To Treat*** | **Sacubitril/ valsartan** | **ACE/ ARB** | **Sacubitril/ valsartan** | **ACE/ ARB** | **HR** | **CRR** |
| HyperK (Any) | 367 | 456 | 276 (249, 305) | 414 (377, 453) | 0.71 (0.62 - 0.81) | 0.75 (0.65 – 0.86) |
| HyperK (Hosp) | 295 | 381 | 214 (190, 239) | 331 (299, 365) | 0.69 (0.59 - 0.80) | 0.72 (0.62 – 0.84) |
| Hypotension (Any) | 246 | 232 | 163 (143, 184) | 169 (148, 191) | 0.99 (0.83 - 1.19) | 1.05 (0.88 – 1.26) |
| Hypotension (Hosp) | 11 | <10** | 6 (3, 11) | 5 (2, 9) | 1.30 (0.52 - 3.23) | 1.37 (0.55 – 3.41) |
| ***As Treated*** | **Sacubitril/ valsartan** | **ACE/ ARB** | **Sacubitril/ valsartan** | **ACE/ ARB** | **HR** | **CRR** |
| HyperK (Any) | 241 | 338 | 299 (263, 339) | 482 (433, 536) | 0.67 (0.57 - 0.80) | 0.71 (0.60 – 0.84) |
| HyperK (Hosp) | 201 | 286 | 242 (210, 278) | 396 (352, 444) | 0.67 (0.56 - 0.80) | 0.70 (0.59 – 0.84) |
| Hypotension (Any) | 131 | 149 | 149 (125, 176) | 177 (150, 207) | 0.90 (0.71 - 1.13) | 0.93 (0.73 – 1.18) |
| Hypotension (Hosp) | <10** | <10** | 4 (1, 10) | 7 (3, 13) | 0.67 (0.19 - 2.38) | 0.70 (0.20 – 2.52) |

(Any) refers to both inpatient and outpatient claims while (Hosp) refers to hospitalization claims only.
Hyperkalemia and Hypotension were defined by ICD9/ICD10 codes.
* Competing risk regression models
** Cells with <10 were censored per USRDS reporting policy.
Abbreviations: CV, cardiovascular; HF, heart failure; ACE/ARB, angiotensin converting enzyme/angiotensin receptor blocker; IR, incidence rate; PY, person years; IRD, incidence rate difference; HR, hazard ratio; CRR, Competing Risk Regression

© 2024 Le D et al. *JAMA Network Open*.

**eFigure 1.** Covariate Balance and Post 1:1 Propensity Score Matching Without Replacement

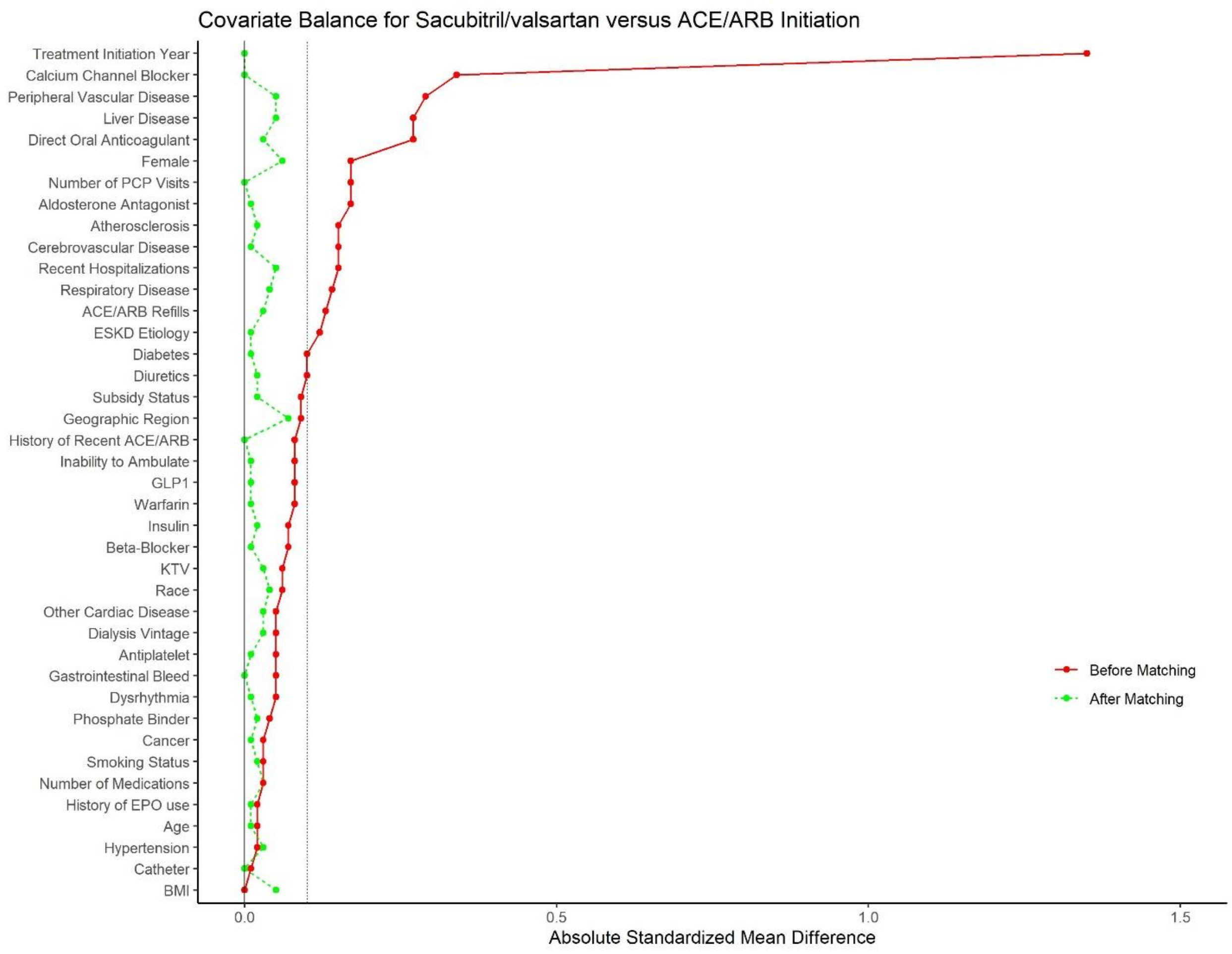


© 2024 Le D et al. *JAMA Network Open*.

**eFigure 2.** Subgroup Analyses of the HR for All-Cause Mortality Comparing Sacubitril-Valsartan vs ACEI/ARB After 1:1 Match Across Demographics, Medical Comorbidities, and Medication Dispensed History

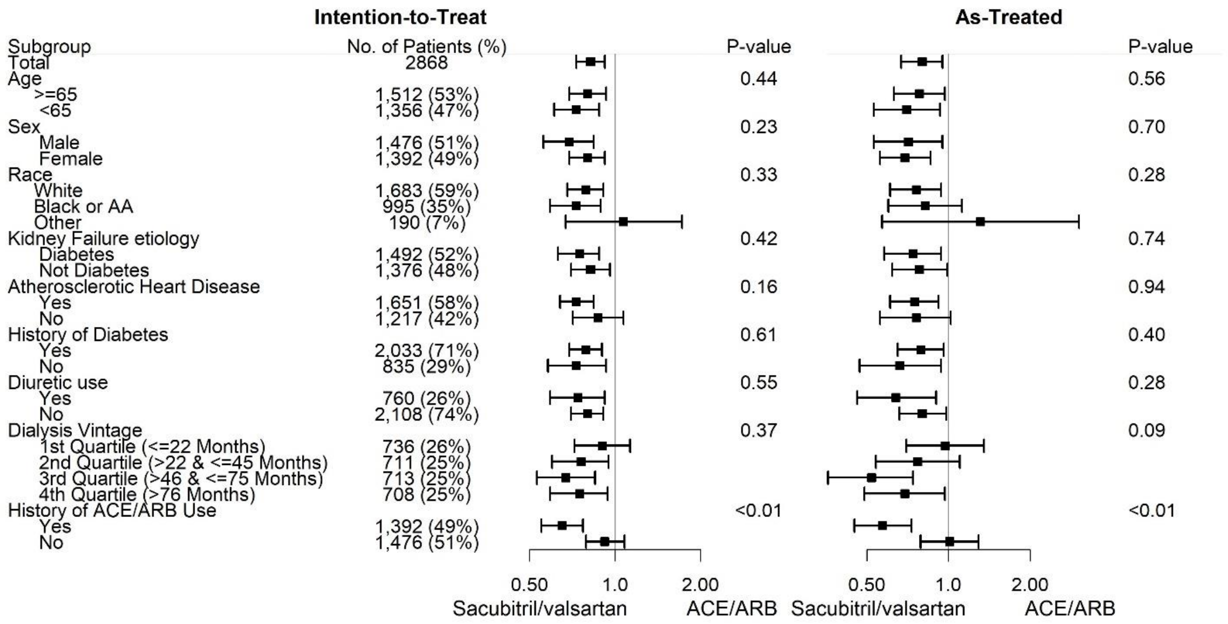


Covariates with poor balance (SMD > 0.10) were included as regression coefficients in the cox-proportional hazards model. P-value from test for interaction.

Abbreviations: AA, African American; ACE/ARB, Angiotensin Converting Enzyme inhibitor/Angiotensin Receptor Blocker

© 2024 Le D et al. *JAMA Network Open*.

**eFigure 3.** Subgroup Analyses of the HR for All-Cause Hospitalization Comparing Sacubitril-Valsartan vs ACEI/ARB

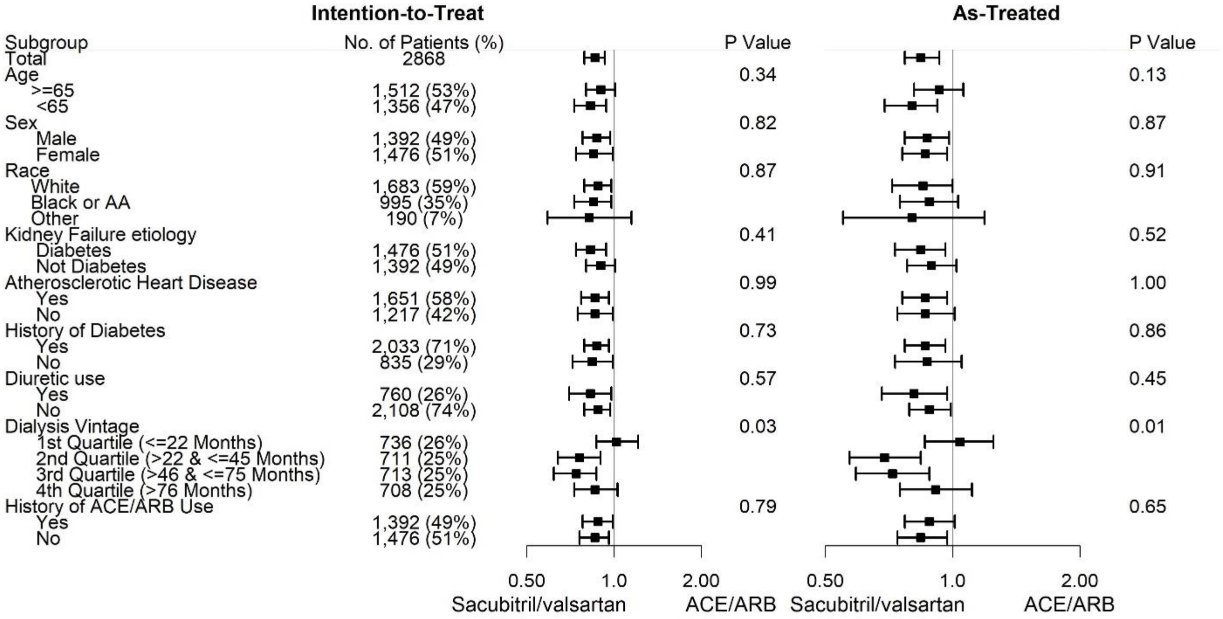


Post 1:1 propensity score match across demographics, medical comorbidities, and medication dispense history. Covariates with poor balance (SMD > 0.10) were included as regression coefficients in the cox-proportional hazards model. P-value from test for interaction.

Abbreviations: AA, African American; ACE/ARB, Angiotensin Converting Enzyme inhibitor/Angiotensin Receptor Blocker

© 2024 Le D et al. *JAMA Network Open*.

**eFigure 4.** Dose-Response Relationship Comparing Both All-Cause Mortality and All-Cause Hospitalization With Sacubitril-Valsartan Initiation Dose

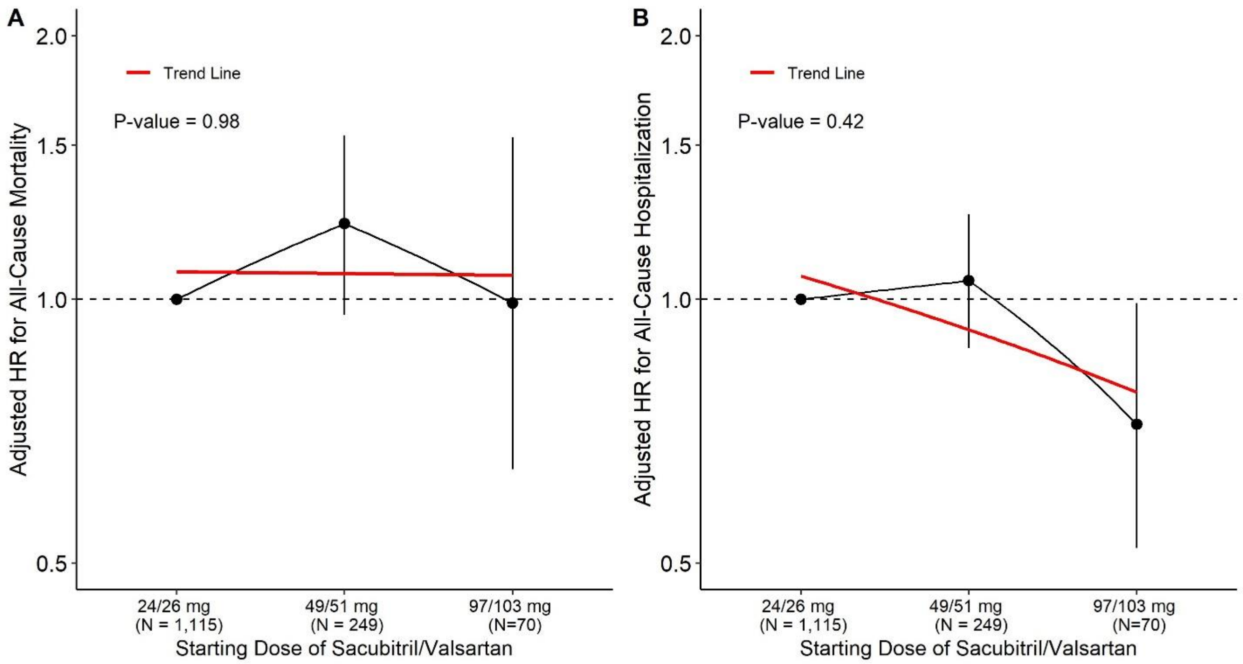


"24/26 mg" is reference. All-cause mortality (**A**) and all-cause hospitalization (**B**). We plot the adjusted cox regression HR with a linear test for trend across the dose range.

© 2024 Le D et al. *JAMA Network Open*.

**eFigure 5.** Sacubitril/Valsartan Dosing Pattern During Follow-Up Defined Using the Starting Dispenses Dose, Maximum Dispensed Dose, and Most Recent Dispensed Dose

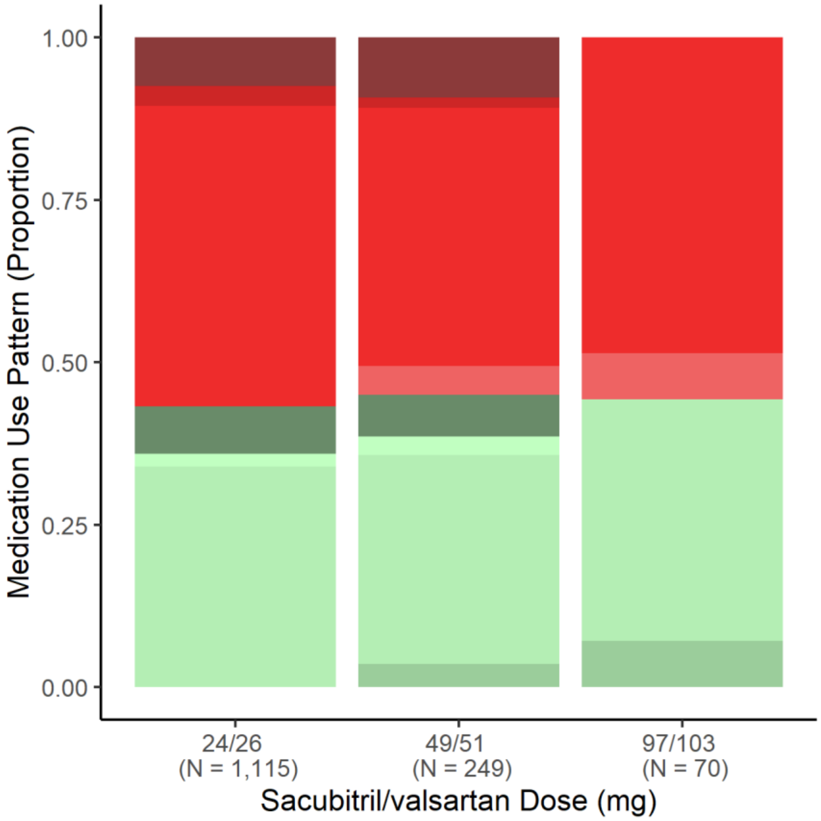


| Medication Use Pattern | Discontinued | Dose Pattern |
|---|---|---|
| Discontinued After Increase | Yes | Start < max & max = final |
| Discontinued After Failed Titration | Yes | Start < max & max > final |
| Discontinued Starting Dose | Yes | Start = max & max = final |
| Discontinued After Decrease | Yes | Start = max & max > final |
| Increased | No | Start < max & max = final |
| Failed Titration | No | Start < max & max > final |
| Stayed on Starting Dose | No | Start = max & max = final |
| Decreased | No | Start = max & max > final |

X-axis values represent the initial dispense dose. Discontinuation was defined as 30 days after end of last dispense without another dispense. Percentages not shown due to some groups having <10 individuals per USRDS reporting policy.

© 2024 Le D et al. *JAMA Network Open*.

© 2024 Le D et al. *JAMA Network Open*.
