# Supplementary material for: Sacubitril-Valsartan in Patients Requiring Hemodialysis
Source: JAMA Netw Open. 2024 Aug 20;7(8):e2429237. doi: 10.1001/jamanetworkopen.2024.29237 (PMC11337068; doi:10.1001/jamanetworkopen.2024.29237)
Supplement: Supplement 2. — Data Sharing Statement [file jamanetwopen-e2429237-s002.pdf]

Data Sharing Statement

Le. Sacubitril-Valsartan in Patients Requiring Hemodialysis. *JAMA Netw Open*. Published August 20, 2024. doi:10.1001/jamanetworkopen.2024.29237

Data
Data available: No

Additional Information
Explanation for why data not available: Data is available and provided by the USRDS via data use agreement.
